# Retrospective evaluation of acute kidney injury in paediatric COVID-19 patients: a tertiary referral hospital experience

**DOI:** 10.1007/s40620-024-01986-9

**Published:** 2024-07-20

**Authors:** Fatma Yazılıtaş, Evrim Kargın Çakıcı, Tülin Güngör, Deniz Karakaya, Evra Çelikkaya, Zeynep Savaş Şen, Rüveyda Gümüşer, Naciye Gönül Tanır, Mehmet Bülbül

**Affiliations:** Dr Sami Ulus Kadin Dogum Cocuk Sagligi ve Hastaliklari Egitim ve Arastirma. Hastanesi, Ankara, Turkey

**Keywords:** Acute kidney injury, Children, COVID-19, Coronavirus, SARS-CoV-2

## Abstract

**Background:**

Coronavirus disease 2019 (COVID-19) has been recognised as a risk factor for acute kidney injury (AKI). Our aim was to investigate the risk factors contributing to hospitalised and outpatient paediatric COVID-19-associated AKI.

**Methods:**

A retrospective observational study was conducted on patients aged 1 month to 18 years with diagnosed COVID-19-associated AKI applied to a tertiary paediatric referral hospital between March 1, 2020 and March 1, 2022.

**Results:**

A total of 6683 patients were evaluated and 486 patients were included in the study. Acute kidney injury was observed in 3.7% of outpatients and 23.9% of hospitalised patients. Multivariate logistic regression analysis showed that, on admission, a history of contact with a COVID-19 positive person (*p* < 0.001), age below 12 months (*p* = 0.004), presence of comorbidities (*p* < 0.001), abdominal pain (*p* = 0.008), anorexia (*p* = 0.003), dyspnoea (*p* = 0.005), higher lactate dehydrogenase values (*p* = 0.004), neutrophilia (*p* < 0.001), higher neutrophil-to-lymphocyte ratio (NLR) (*p* = 0.003), higher white blood cell counts (*p* = 0.006), elevated C-reactive protein (CRP) levels (*p* = 0.002), anaemia (*p* = 0.015), hypoalbuminaemia (*p* < 0.001), hyperglycaemia (*p* = 0.006), and presence of proteinuria (*p* = 0.003) were independent predictors of AKI. Higher rates of hospitalisation (*p* < 0.001) and admission to the paediatric intensive care unit (PICU) (*p* < 0.001), longer length of hospitalisation (*p* < 0.001), and greater need for mechanical ventilation (*p* < 0.001) were associated with AKI.

**Conclusions:**

This study reveals that not only hospitalised children, but also paediatric patients are at risk for AKI. The presence of comorbidities, abdominal pain, anorexia, dyspnoea, anaemia, inflammation, hypoalbuminaemia, proteinuria and history of contact with a COVID-19 positive person were the main risk factors for AKI. COVID-19-associated AKI was associated with worse outcomes.

**Graphical abstract:**

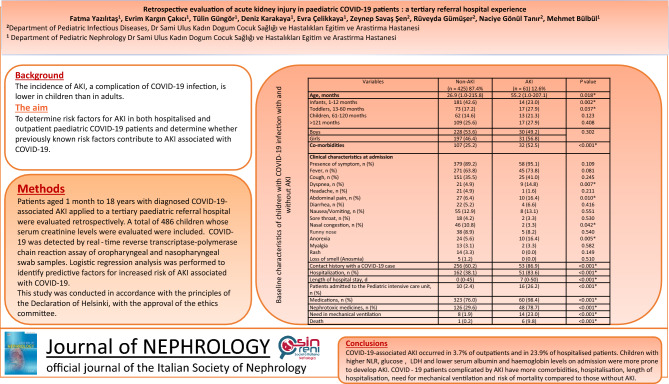

## Introduction

The novel severe acute respiratory syndrome coronavirus 2 (SARS-CoV-2), known as the cause of coronavirus disease 2019 (COVID-19), was first identified in Wuhan, China in December 2019 [1]. The presentation of COVID-19 varies widely, ranging from an asymptomatic response, to isolated respiratory and/or gastrointestinal symptoms, or to the development of multiorgan systemic disease [2]. There is evidence that the kidney is a target organ for SARS-CoV-2, and that the highly expressed angiotensin-converting enzyme 2 receptor on renal tubular cells and podocytes are required for SARS [3]. Acute kidney injury (AKI) is associated with COVID-19 severity and outcome, and seems to be a risk factor for adverse outcomes among both outpatients and hospitalised children [1, 4]. The reported incidence of AKI in children with COVID-19 ranges from 0 to 70% [5]. There are limited data regarding AKI-associated risk factors and potential outcomes in children with COVID-19. It has been reported that exposure to nephrotoxic drugs, high C-reactive protein (CRP), increased white blood cell (WBC) counts, and low serum albumin levels may be risk factors for AKI in children with COVID-19 [2, 3, 6]. The inflammatory state itself may be a manifestation of COVİD-19 infection that predisposes to renal hypoperfusion and AKI. Despite these data, the risk factors and potential outcomes of AKI associated with COVID-19 in paediatric patients are not well defined. Estimation of the frequency of AKI and identification of risk factors in children with COVID-19 remain essential to guide the treatment of these patients. Although risk factors for AKI have been reported in hospitalised patients, the factors for identifying patients at high risk of AKI in outpatient settings are unknown and may differ from those recorded in hospitalised children.

We aimed to describe risk factors for AKI in both hospitalised and outpatient paediatric COVID-19 patients and determine if previously known risk factors contribute to AKI associated with COVID-19.

## Methods

### Patients and data collection

In this retrospective observational study, 6683 children with a polymerase chain reaction (PCR)-confirmed diagnosis of COVID-19 infection admitted to the COVID-19 polyclinic or emergency department of a tertiary paediatric referral hospital from March 1, 2020 to March 1, 2022 were selected. Among them, serum creatinine values were evaluated in 486 patients due to their clinical conditions, and they were included in the study. Data regarding demographics and clinical characteristics, laboratory findings, radiologic investigations, hospital stay, and medication use during hospitalisation were collected from the hospital electronic records. The patients were divided into two groups i.e., AKI and non-AKI.

Baseline and peak serum creatinine levels, baseline and the lowest estimated glomerular filtration rate (eGFR), kidney and liver function tests, electrolytes, serum levels of blood urea nitrogen (BUN), serum albumin, lactate dehydrogenase (LDH), complete blood count, the highest CRP, ferritin, creatine kinase (CK), d-dimer, troponin values, and urine dipstick tests, urine output (UOP), urine culture and blood culture were recorded (if available). Echocardiography (ECHO), chest X-ray and computed tomography were performed when indicated.

### Inclusion criteria

The inclusion criteria were: (1) patients aged between 1 month to 18 years with a confirmed diagnosis of COVID-19 detected by real-time reverse transcriptase-polymerase chain reaction (RT-PCR) assay of oropharyngeal and nasopharyngeal swab samples; (2) patients whose serum creatinine levels and eGFRs were evaluated during the follow-up period.

### Exclusion criteria

Patients older than 18 years of age and neonates (aged 0–28 days at presentation) were excluded from the study. We also excluded patients with a known pre-existing history of chronic kidney disease and end-stage kidney disease (ESKD), and patients whose serum creatinine levels and medical history were unknown.

### Definitions

COVID-19 infection was defined as real-time RT-PCR-positive oropharyngeal and nasopharyngeal swabs. An infected person (testing positive for COVID-19 via PCR testing) was defined as being COVID-19 positive.

The modified Schwartz formula to determine eGFR was used [7].

Using the “Kidney Disease: Improving Global Outcomes” (KDIGO) guidelines, AKI was defined as an increase of serum creatinine by ≥ 0.3 mg/dl within 48 h; or known/presumed increase from the baseline creatinine by ≥ 50% within the prior 7 days; or a urine output < 0.5 mL/kg/hours for 6 h [8]. Staging of AKI was defined according to KDIGO guidelines. For outpatients whose urine output could not be reliably collected due to difficulty of data capture of urine output, only an increase in serum creatinine was used as the definition of AKI.

Baseline serum creatinine was defined as the lowest serum creatinine level within the previous 3 months before admission [9]. In addition, in patients without previous creatinine values, the lowest creatinine value obtained at COVID-19 disease presentation was defined as the baseline value.

Proteinuria was defined as protein/creatinine ratio greater than 0.2 mg/mg in spot urine test, or higher than 4 mg/m^2^/hour in 24-h urine, or ≥ 1 + protein on urine dipstick test [10].

The absolute neutrophil count was divided by the absolute lymphocyte count to calculate the neutrophil-to-lymphocyte ratio (NLR).

### Outcomes

The influence of AKI on clinical course and outcomes was also taken into consideration. The outcomes included were hospitalisation, length of hospitalisation, need for kidney replacement therapy (KRT), mechanical ventilation, admission to the paediatric intensive care unit (PICU), and mortality.

### Statistical analysis

All analyses were performed using the statistical software package (SPSS) for Windows (version 21.0). Continuous data are expressed as means ± standard deviations, and categorical data are given as frequencies and percentages. Non-normally distributed data were expressed as median with data range (minimum to maximum). Logistic regression analysis was performed to identify predictive factors for increased risk of AKI associated with COVID-19. Data were expressed as odds ratios (OR) with 95% confidence intervals (CI). Between-group comparisons for continuous and categorical variables were performed by the Mann–Whitney U test and the chi-square test, respectively. Multivariate analysis was performed with parameters found to be significant in the evaluation of univariate analysis, and predictive values of variables were calculated to assess the relationship between AKI and outcomes. A *p*-value less than 0.05 was accepted as statistically significant.

## Results

### Incidence of AKI

**Fig. 1:**
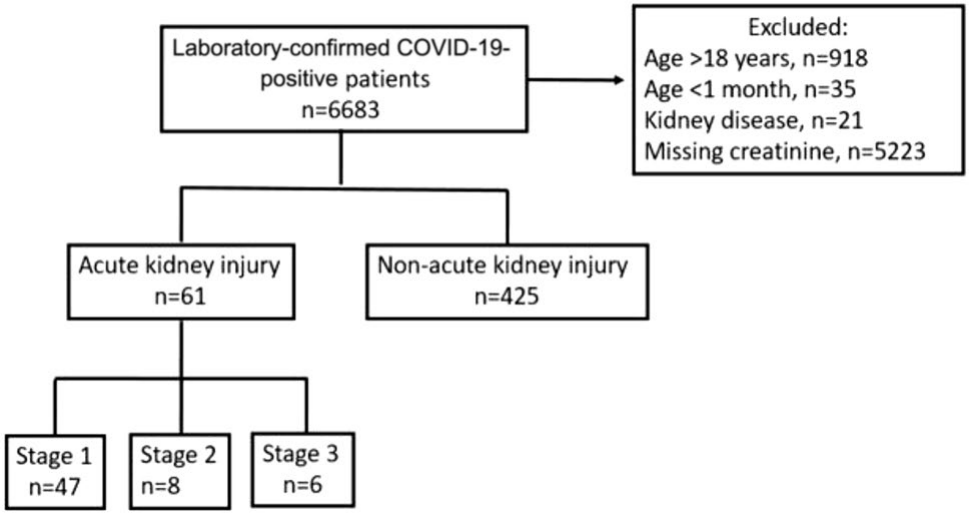
Flowchart of study population

A total of 6683 patients admitted to our hospital with a PCR-confirmed diagnosis of COVID-19 infection during the study period were evaluated, and 486 children whose serum creatinine levels were assessed were included in the study (Fig. 1). Acute kidney injury was observed in 10 (3.7%) outpatients and in 51 (23.9%) hospitalised patients.

### Clinical characteristics of patients

**Fig. 2:**
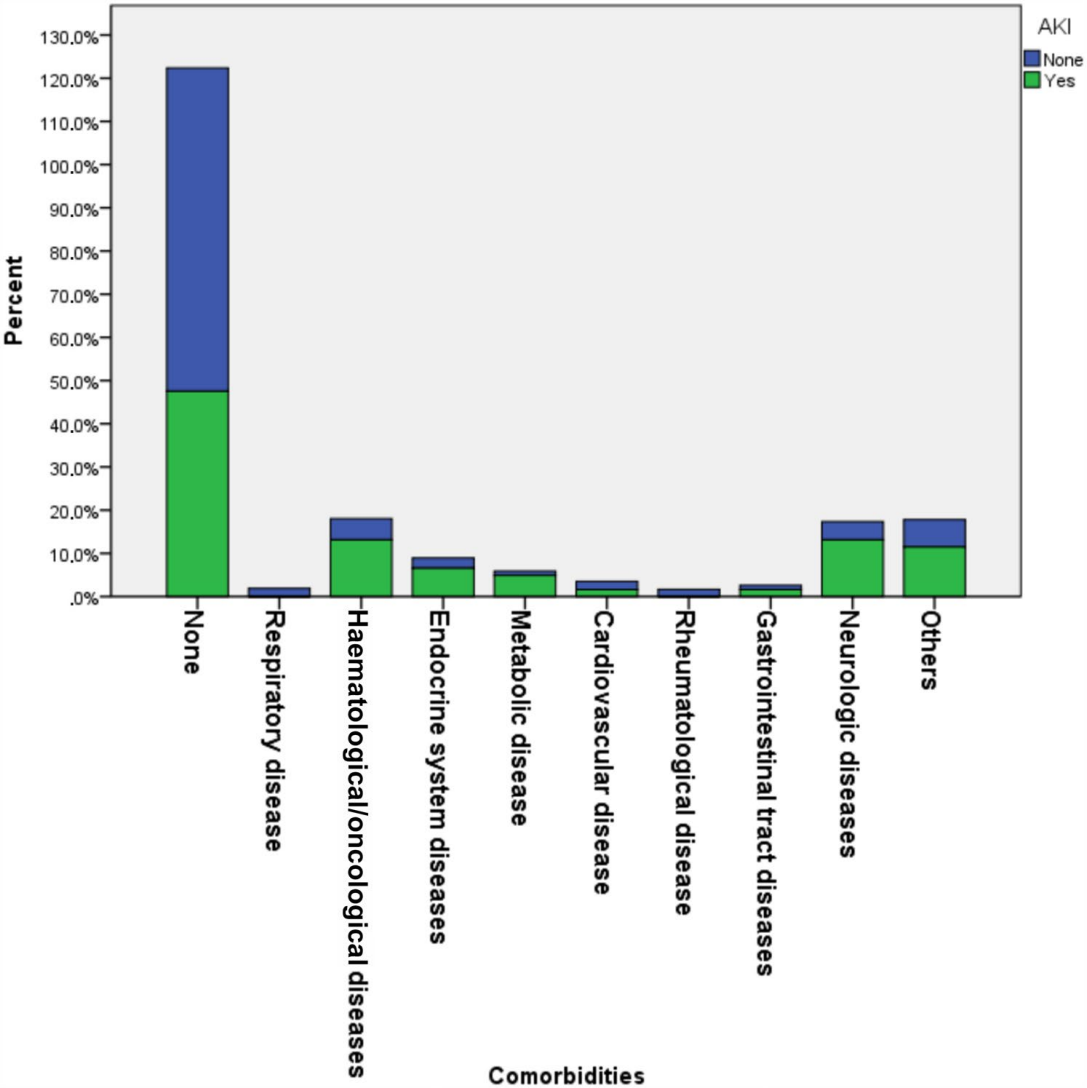
Patient’s comorbidities. Bar chart showing percentages of patients with various underlying comorbidities

The median age of the patients was 31.7 (range 1.0–215.8) months. Patients with AKI were older than those without [55.2 (range 1.0–207.1) vs. 26.9 (range 1.0–215.8) months]. The majority of patients (63.6%) had had contact with a person who was COVID-19 positive. Fever (*n* = 316, 65.0%) was the most common presenting symptom of COVID-19-positive patients, while some patients (*n* = 49, 10.1%) were asymptomatic. Comorbidity was present in 28.6% of the patients, and patients with AKI had more comorbidities compared to those without AKI (*p* < 0.001) (Fig. 2; Table 1).

**Table 1:** Baseline characteristics of children with and without AKI

Variables	Non-AKI *n* = 425 (87.4%)	AKI *n* = 61 (12.6%)	*p* value
Age, months, median (min -max)	26.9 (1.0–215.8)	55.2 (1.0–207.1)	0.249
*Age category*, *n* (%)			
Infants, 1–12 months	181 (42.6)	14 (23.0)	0.002*
Toddlers, 13–60 months	73 (17.2)	17 (27.9)	0.037*
Children, 61–120 months	62 (14.6)	13 (21.3)	0.123
Adolescents, > 121 months	109 (25.6)	17 (27.9)	0.408
*Gender*, *n* (%)			0.302
Male	228 (53.6)	30 (49.2)	
Female	197 (46.4)	31 (56.8)	
Contact history with a COVID-19 patient, *n* (%)	256 (60.2)	53 (86.9)	< 0.001*
*Clinical characteristics at admission*, *n* (%)			
Presence of symptoms	379 (89.2)	58 (95.1)	0.109
Fever	270 (63.5)	46 (75.4)	0.045*
Cough	151 (35.5)	25 (41.0)	0.245
Dyspnoea	21 (4.9)	9 (14.8)	0.007*
Headache	21 (4.9)	1 (1.6)	0.211
Abdominal pain	27 (6.4)	10 (16.4)	0.010*
Diarrhoea	22 (5.2)	4 (6.6)	0.416
Nausea/Vomiting	55 (12.9)	8 (13.1)	0.551
Sore throat	18 (4.2)	2 (3.3)	0.530
Nasal congestion	46 (10.8)	2 (3.3)	0.042*
Runny nose	38 (8.9)	5 (8.2)	0.540
Anorexia	24 (5.6)	10 (16.4)	0.005*
Myalgia	13 (3.1)	2 (3.3)	0.582
Rash	14 (3.3)	0 (0.0)	0.149
Loss of smell (Anosmia)	5 (1.2)	0 (0.0)	0.510
*Comorbidities*, *n* (%)			
Presence of comorbidity	107 (25.2)	32 (52.5)	< 0.001*
Cardiovascular disease	15 (3.5)	5 (8.2)	0.092
Endocrine system diseases	12 (2.8)	9 (14.8)	< 0.001*
Gastrointestinal tract diseases	6 (1.4)	2 (3.3)	0.265
Haematological-oncological diseases	21 (4.9)	8 (13.1)	0.020*
Metabolic disease	4 (0.9)	4 (6.6)	0.011*
Neurologic diseases	23 (5.4)	12 (19.7)	< 0.001*
Rheumatological disease	7 (1.7)	0 (0.0)	0.398
Respiratory disease	9 (2.1)	1 (1.6)	0.636
Others	35 (8.2)	15 (24.6)	< 0.001*
Hospitalisation, *n* (%)	162 (38.1)	51 (83.6)	< 0.001*
*Patients admitted to PICU*, *n* (%)	10 (2.4)	16 (26.2)	< 0.001*
Medications, *n* (%)			
None	102 (24.0)	1 (1.6)	< 0.001*
Single medication	189 (44.5)	14 (23.0)	0.001*
Multiple medications	134 (31.5)	46 (75.4)	< 0.001*
Nephrotoxic medications**	126 (29.6)	48 (78.7)	< 0.001*
Need for mechanical ventilation, *n* (%)	8 (1.9)	14 (23.0)	< 0.001*
Mortality, *n* (%)	1 (0.2)	6 (9.8)	< 0.001*

*AKI* Acute Kidney Injury, *PICU* Paediatric Intensive Care Unit

*Statistically significant *p* values

**Nephrotoxic medications include aminoglycosides, trimethoprim/sulfamethoxazole, vancomycin, acyclovir, nonsteroidal anti-inflammatory drugs, antiviral agents, methotrexate, phenytoin, levetiracetam, angiotensin-converting enzyme, and angiotensin-receptor blockers

### Clinical course and outcomes

Acute kidney injury was significantly associated with a greater likelihood of requiring hospitalisation (83.6% vs. 38.1%, *p* < 0.001). Acute kidney injury was significantly associated with admission to the PICU (26.2% vs. 2.4%, *p* < 0.001), need for mechanical ventilation (23.0% vs. 1.9%, *p* < 0.001), and mortality (9.8% vs. 0.2%, *p* < 0.001) in paediatric patients with COVID-19. Demographic data and admission characteristics of paediatric patients with COVID-19 with/without AKI diagnosis are presented in Table 1.

Patients with AKI had significantly higher levels of serum glucose, alkaline phosphatase, lactate dehydrogenase, ferritin, CRP, white blood cell and neutrophil count compared to patients without AKI. In addition, the levels of serum albumin, calcium and haemoglobin on admission were significantly lower in patients who developed AKI (Table 2). Patients with AKI had significantly more proteinuria when compared with patients without AKI (10.5% vs. 1.8%, *p* = 0.022), however, proteinuria was not associated with fever symptoms (*p* = 0.261). Furthermore, proteinuria was not detected in patients younger than 24 months of age.

**Table 2:** Laboratory data of children with COVID-19 infection with and without AKI

Variables	AKI *n* = 425 (87.4%)	AKI *n* = 61 (12.6%)	*p* value
Blood urea nitrogen, mg/dL, mean ± SD	9.0 ± 3.9	9.3 ± 4.7	0.360
Uric acid, mg/dL, mean ± SD	3.9 ± 1.9	3.9 ± 1.5	0.392
Glucose, mg/dL, mean ± SD	97.8 ± 20.3	119.6 ± 57.1	0.003*
Albumin, g/dL, mean ± SD	4.1 ± 0.4	3.6 ± 0.5	< 0.001*
Calcium, mg/dL, mean ± SD	9.7 ± 0.6	8.9 ± 1.0	< 0.001*
Alkaline phosphatase, IU/L, (median, min.–max.)	137.5 (35–449)	202.5 (29–952)	0.014*
Lactate dehydrogenase, IU/L, mean ± SD	244.2 ± 96.4	403.5 ± 153.8	< 0.001*
Creatine kinase, IU/L, (median, min.–max.)	87.5 (14.0–316.0)	68.0 (12.0–364.0)	0.792
D-Dimer ng/mL FEU, (median, min.–max.)	760.5 (13–1875)	1901.5 (0.3–24,776)	0.240
Troponin (ng/mL), (median, min.–max.)	0.0 (0.0–2.8)	0.0 (0.0–0.3)	0.435
C-Reactive protein, mg/L	2.9 (0.1–136,6)	14.8 (0.1–272.9)	0.005*
Ferritin, ng/mL, (median, min.–max.)	22.0 (1.6–362.0)	834.0 (28.5–3397.1)	0.018*
*Complete blood cell count*			
WBC count, × 10^3^/μL, (median, min.–max.)	6.7 (0.7–32.0)	7.8 (0.3–53.3)	0.002*
Neutrophil count, × 10^3^/μL, (median, min.–max.)	3.5 (0.4–26.0)	7.5 (0.5–27.5)	< 0.001*
Lymphocyte count, × 10^3^/μL, (median, min.–max.)	2.3 (0.2–12.5)	2.1 (0.1–6.7)	0.181
Haemoglobin, g/dL, mean ± SD	12.4 ± 1.7	11.5 ± 2.3	0.012*
Platelet count, × 10^3^/μL, mean ± SD	291.4 ± 101.0	263.1 ± 150.1	0.216
NLR, (median, min.–max.)	1.4 (0.2–14.4)	4.1 (0.2–23.1)	0.001*

*AKI* Acute Kidney Injury, *SD* Standard Deviation, *eGFR* estimated Glomerular Filtration Rate, *WBC* White Blood Cell, *NLR* Neutrophil-to-Lymphocyte Ratio

*Statistically significant *p* values

### Severity of AKI

Patients with AKI were staged according to the 2012 KDIGO guidelines and were stratified as follows: stage I, *n* = 47 (77.1%); stage II, *n* = 8 (13.1%); and stage III, *n* = 6 (9.8%). Three patients with AKI stage III required KRT. The medications administered to patients during hospitalisation included nephrotoxic drugs, bronchodilators, antihypertensive agents, antiepileptics, corticosteroids, intravenous immunoglobulin, vasopressors, and anticoagulant or antiplatelet drugs. The proportion of patients receiving vasopressor drugs in the AKI group was significantly higher than in the non-AKI group (9 versus 1, *p* < 0.001). Those treated with corticosteroids and antivirals were higher in the AKI group than in the non-AKI group (21 versus 16, and 11 versus 5, respectively [*p* > 0.05]). Also, there was no significant difference between AKI stage and use of medications during hospitalisation (*p* > 0.05), except for vasopressor drugs. In addition, no statistically significant relationship was found between AKI stage and the need for hospitalisation, length of hospital stay, and admission to the PICU and comorbidities (*p* > 0.05). However, a greater percentage of stage III AKI patients had higher mortality rates [stage I: 4.3% vs. stage II: 0.0% vs. stage III: 66.7%; *p* < 0.001] and need for mechanical ventilation [stage I: 19.1% vs. stage II: 12.5% vs. stage III: 66.7%; *p* = 0.025].

### Risk factors for AKI

Multivariate logistic regression analysis revealed that on admission a history of contact with a COVID-19 positive person (*p* < 0.001), age younger than 12 months (*p* = 0.004), presence of underlying diseases (*p* < 0.001), abdominal pain (*p* = 0.008), anorexia (*p* = 0.003), and dyspnoea (*p* = 0.005) were significantly predictive risk factors for AKI. Also, multivariate analysis showed that neutrophilia (*p* < 0.001), anaemia (*p* = 0.015), hypoalbuminaemia (*p* < 0.001), hyperglycaemia (*p* = 0.006), and presence of proteinuria (*p* = 0.003) were independent predictors of AKI. The levels of white blood cells (*p* = 0.006), CRP (*p* = 0.002), NLR (*p* = 0.003), and lactate dehydrogenase (*p* = 0.004) were significantly higher in patients with AKI compared to those without AKI (Table 3).

**Table 3:** Independent risk factors for AKI by multivariable analysis

Variables	Multivariate regression analysis		
	OR	95% CI	*p* value*
Age from 1 to 12 months old	0.40	0.2–0.7	0.004
Contact history with a COVID-19 case	4.37	2.0–9.4	< 0.001
Abdominal pain	2.89	1.3–6.3	0.008
Anorexia	3. 72	1.4–7.2	0.003
Dyspnoea	3.33	1.4–7.6	0.005
Presence of comorbidities	3.27	1.8–5.6	< 0.001
Glucose	1.02	1.0–1.1	0.006
Albumin	0.11	0.1–0.2	< 0.001
Calcium	0.29	0.1–0.5	< 0.001
Lactate dehydrogenase	1.01	1.0–1.1	0.004
Haemoglobin	0.79	0.6–0.9	0.015
White Blood Cell count	1.07	1.0–1.1	0.006
Neutrophil count	1.15	1.1–1.2	< 0.001
Neutrophil to lymphocyte ratio	1.22	1.1–1.4	0.003
C-Reactive protein	3.00	1.5–5.9	0.002
Presence of proteinuria	9.87	2.1–45.2	0.003
Hospitalisation	7.37	3.7–14.5	< 0.001
Length of hospital stay	1.20	1.1–1.3	< 0.001
Medications	18.94	2.5–138.4	0.004
Paediatric intensive care unit	14.72	6.3–34.3	< 0.001
Need for mechanical ventilation	15.48	6.1–38.8	< 0.001
Mortality	46.14	5.4 -390.4	< 0.001

*OR* odds ratios, *CI* Confidence Intervals

*Statistically significant *p* values

Higher rates of hospitalisation (OR 7.37, 95% CI, 3.7–14.5, *p* < 0.001) and admission to the PICU (OR 14.72, 95% CI, 6.3–34.3, *p* < 0.001), longer length of hospitalisation (OR 1.20, 95% CI, 1.1–1.3, *p* < 0.001), use of more medications during hospitalisation (OR 18.94, 95% CI, 2.5–138.4, p = 0.004), and greater need for mechanical ventilation (OR 15.48, 95% CI, 6.1–38.8, *p* < 0.001) were associated with AKI (Table 3).

## Discussion

There have been relatively few studies focusing on AKI in children [8, 11]. Although a high incidence of AKI has been reported as a prominent feature in adult COVID-19 infection [2, 12, 13], a lower incidence of AKI in children has been shown [8, 11, 14]. One of the studies reporting a lower rate of AKI was a retrospective study conducted on 238 paediatric hospitalised patients and outpatients in China, in whom AKI was present in only 3 patients (1.2%) [14], much lower than that reported in the United States (24%) [11], and in the United Kingdom (29%) [15]. A meta-analysis reported that 44% of critically ill children (a total of 106) developed AKI according to the KDIGO serum creatinine criteria [16]. The discrepancies in the frequency of COVID-19-associated AKI among countries may be explained by different inclusion criteria (age, severity of illness, hospitalised or outpatients, intensive care admissions, and different definitions of AKI), regional differences, and different demographic characteristics of the study populations [17].

Although conflicting results have been reported, comorbidities generally increase the severity of AKI in patients with COVID-19. The presence of comorbidities, such as respiratory diseases, cardiac diseases, haematological disorders, and malignancies are likewise important in children with COVID-19 who developed AKI [6, 19–21]. A systematic review reported the rate of underlying comorbid disease in children with AKI as 26.7% [21]. Our patients showed a similar prevalence of comorbidities (28.6%) and 52.5% of patients with AKI had underlying diseases (Table 1). A higher prevalence of comorbidities has been reported in adult patients with COVID-19-associated AKI [18]. By comparison with adults, paediatric patients had a lower prevalence of, and different comorbidities. COVID-19 and AKI may lead to worsening of comorbid disease [1]. However, we observed similar comorbidities in COVID-19 patients with and without AKI (*p* > 0.05). Providing early treatment may be an explanation for our positive results. A child with a comorbid disease is usually overprotected by parents and may have been brought to medical attention earlier than others, which might have reduced kidney injury by allowing timely medical interventions.

Various mechanisms are possibly involved in kidney injury during COVİD-19 infection, including direct infection of the kidney parenchyma by the virus, endothelial injury, ischaemic acute tubular necrosis, Renin–Angiotensin–Aldosterone System imbalance, microthrombosis, increased vascular permeability, and volume depletion, as well as the development of kidney damage secondary to haemodynamic instability, inflammatory cytokines, and therapeutic approaches (nephrotoxic medications, mechanical ventilation) [3, 6, 11, 22]. Studies have shown that the aetiologies of AKI in patients with COVID-19 seem to be multifactorial, which is also consistent with our results. A meta-analysis reported that fever and gastrointestinal symptoms, were more common in children with AKI associated with COVID-19 [3, 6, 11]. The present study showed that the main risk factors for the development of AKI were older age and the presence of abdominal pain, dyspnoea, fever, nasal congestion or anorexia as initial symptoms. All of these symptoms may lead to decreased fluid intake and increased fluid loss, which are triggering factors for the development of AKI in patients. Young children, and especially infants, with COVID-19 are more prone to develop gastrointestinal symptoms of COVID-19, and dehydration significantly increases the risk of AKI [14]. Haemodynamic monitoring with appropriate fluid management and vasopressor drugs are required to provide adequate renal perfusion in AKI patients. In our study, the need for vasopressors was higher in AKI patients (*p* < 0.001). We observed that AKI was associated with abdominal pain, but not nausea, vomiting and diarrhoea in our patients. These unexpected results can be explained by the fact that the amount of vomiting and diarrhoea in our patients was not high enough to cause fluid loss. Due to the retrospective nature of our study, the frequency and amount of both vomiting and diarrhoea are unknown.

It has been suggested that the systemic pro-inflammatory response may play an important role in the development of AKI in COVID-19 patients [6, 11, 17, 22]. Similarly, in our study, increased WBC and neutrophil count, increased NLR, increased serum levels of CRP and ferritin, and decreased serum albumin levels were found to be statistically significant for AKI patients compared to patients without AKI. The lower serum albumin and consequently lower serum calcium levels we detected in our patients with AKI may be explained by increased capillary permeability secondary to systemic inflammation.

Proteinuria was detected in 10.5% of our AKI patients by urinalysis and AKI was significantly associated with the presence of proteinuria (*p* = 0.022). Although it is known that febrile episodes can cause transient proteinuria in children, we could not show any difference in proteinuria between the patients with or without fever (*p* = 0.261). On the other hand, unlike other infections that cause transient proteinuria [10], COVID-19 may act directly on the kidney through multiple mechanisms [3, 22].

The present study confirmed that AKI was a risk factor for admission to hospital and the paediatric ICU, and that patients with AKI were more likely to require longer hospital stays. The association between AKI and admission to the ICU, and hospital length of stay has also been reported by other paediatric studies [6, 11, 19]. This association may be explained by haemodynamic instability and a diagnosis of AKI. In the present study, we did not find a statistically significant relationship between AKI stage and hospitalisation requirement, admission to the ICU and length of hospital stay. It may be speculated that the medical care or attention given to patients with COVID-19 could positively influence the outcomes of patients who receive an early diagnosis of AKI. It may also reflect the early management of transient renal ischaemia, which can cause an increase in serum creatinine. This may also be explained by our small sample size.

Mechanical ventilation and nephrotoxic medications were reported as risk factors for AKI development in children with COVİD-19 in a retrospective chart review of children [6, 11, 19].

In the present study, most AKI patients were critically ill and required mechanical ventilation and multiple medications. These results were also consistent with already reported AKI findings in seriously ill children [23]. In this study, there was no significant difference between AKI and use of medications, except for vasopressor drugs. Nephrotoxic medications (i.e., nonsteroidal anti-inflammatory drugs, aminoglycosides, trimethoprim/sulfamethoxazole, vancomycin and acyclovir) are commonly used in most cases. Early identification of AKI and supportive therapy may have helped in the rapid recovery of patients with COVID-19 and AKI, and may also have the potential to decrease the use of nephrotoxic medications. Efficient anti-inflammatory therapy makes prompt renal recovery possible. A beneficial effect of anticoagulant therapy on reducing COVID-19 mortality has been reported [24].

Acute kidney injury has a significant effect on mortality in COVID-19 patients, and higher AKI stage is associated with greater mortality. COVID-19 patients with AKI had a significantly higher risk of mortality compared to COVID-19 patients without AKI [11, 19, 24]. We found a mortality rate of 1.4% in COVID-19-positive patients. This was lower than the AKI mortality rate of 3.3% reported in the literature regarding patients with COVID-19 [25].

Our study has several limitations. Firstly, a baseline serum creatinine measurement was unavailable as most of our COVID-19 patients were previously healthy. Also, given the observational nature of this study, information on serum creatinine levels could not be retrieved as it was not usually evaluated in outpatients. Therefore, we cannot generalise our findings for AKI outpatients. The baseline serum creatinine level was unknown for some patients, which may have caused under-reporting of AKI diagnoses. The second limitation of our study is that only PCR-positive confirmed COVID-19 patients were included, and the sensitivity reported in clinical practice ranges from 42 to 83%, depending on the patient’s symptom duration and viral load, and the quality of the test sample. Thirdly, we may have missed asymptomatic or mildly symptomatic patients treated at home. The lack of examination on tubular proteinuria is a further limitation of this study. Despite these limitations, this study draws its strength from the inclusion of AKI patients in both outpatient and inpatient settings [26].

In conclusion, AKI was diagnosed in 3.7% of outpatients and in 23.9% of hospitalised paediatric patients with COVID-19. Our findings highlight that children with higher levels of inflammation markers, particularly a higher NLR and lower serum albumin levels on admission may be more prone to develop COVID-19-associated AKI. In the present study, COVID-19 patients complicated by AKI had more co-morbidities, lower haemoglobin levels, and higher serum glucose and lactate dehydrogenase levels on admission. We also showed that paediatric COVID-19 patients with AKI requiring hospitalisation had more admissions to the PICU, longer hospital stays, and greater need for mechanical ventilation compared to those without AKI. Patients with COVID-19 AKI had a higher risk of mortality than patients with COVID-19 without AKI. Given the link between COVID-19-associated AKI and both mortality and poor prognosis, the outcome of this study suggests that renal function in children should be closely monitored.
